# One- and two-dimensional electromagnetically induced gratings in an Er^3+^ - doped yttrium aluminum garnet crystal

**DOI:** 10.1038/s41598-020-60809-6

**Published:** 2020-03-04

**Authors:** Tao Shui, Ling Li, Xin Wang, Wen-Xing Yang

**Affiliations:** 1 School of Physics and Optoelectronic Engineering, Yangtze University, Jingzhou, Hubei 434023 China; 2 Department of Physics, Southeast University, Nanjing, 211189 China

**Keywords:** Atomic and molecular interactions with photons, Quantum optics

## Abstract

A coherently prepared Er^3+^-doped yttrium aluminum garnet (YAG) crystal with a four-level ionic configuration is exploited for realizing one-dimensional (1D) and two-dimensional (2D) electromagnetically induced gratings (EIGs). Owing to the probe gain induced by the incoherent pump, the diffraction efficiency of the crystal grating, especially the first-order diffraction, can be significantly improved via increasing the incoherent pumping rate or decreasing the probe detuning. The enhancement of the grating diffraction efficiency originates from the interference between the gain and phase gratings. It is also demonstrated that the diffraction of the crystal grating can be dynamically controlled via tuning the intensity and detuning of the standing-wave driving field or the concentration of Er^3+^ ion. More importantly, the probe energy of the diffraction side lobes around the central principle maximum is comparable to that of the first-order diffraction field for small driving intensity or large driving detuning. Our scheme may provide a possibility for the active all-optical control of optical switching, routing and storage in fiber communication wavelengths.

## Introduction

In the past few decades, the study of electromagnetically induced grating (EIG) has been one of the hot spots in optics due to its potential applications in optical switching and routing^1,2^, optical bistability^3^, light storage^4^, self-imaging^5,6^, and four-wave mixing dipole soliton^7^. Note that EIG, which is created by using a standing-wave (SW) laser field to replace the traveling-wave laser field in electromagnetically induced transparency, can diffract the incident probe beam into high-order diffraction directions. Such a diffraction grating is derived from the spatial periodic modulation of the amplitude and phase of the transmission function. It was first theoretically proposed by Xiao *et al*.^8^ and experimentally observed in cold atomic systems^9,10^. Since then, EIG has been extensively investigated in atomic systems^11–22^, crystal of molecular magnets system^23^, quantum wells and dots^24–27^ and hybrid artificial molecule^28,29^. Among these studies, the improvement of the diffraction efficiency of the coherent grating can be achieved via some feasible approaches such as giant Kerr nonlinearity^11^, Raman gain^14^, parity-time symmetry (or antisymmetry)^18–20,26^, van der Waals interaction^22^ and the surface plasmon and tunneling effect^29^.

On the other hand, much attention has been attracted to the study of Er^3+^-doped yttrium-aluminum-garnet (YAG, chemical formula Y_3_Al_5_O_12_) crystal since the stimulated emission from Er^3+^ ions in the YAG crystal was first observed by Zharikov *et al*.^30^. It should be worth pointing out that Er^3+^-doped YAG crystal, where some of the Y^3+^ ions are replaced by Er^3+^ ions, is an efficient active medium for solid-state lasers operating in the eye-safe wavelengths^31^, which have been applied to the fields of optical communication and biomedicine. Recent years, based on the atomic coherence and quantum interference effects, many kinds of quantum optical phenomena, such as electromagnetically induced transparency (EIT)^32^, large refractive index with vanishing absorption^33^, positive and negative dispersion^34^, flattened gain^35^ and optical bistability and multistability^36,37^, have been studied in Er^3+^-doped YAG crystals. These solid-state systems based on the Er^3+^-doped YAG crystals have the similar properties to atomic vapors, but with the advantage of no atomic diffusion. So far, to our best knowledge, studies have not been extended to the investigation of the diffraction of the EIG in the Er^3+^-doped YAG crystal.

In this paper, we investigate the Fraunhofer diffraction characteristics of one-dimensional (1D) and two-dimensional (2D) diffraction gratings realized in an Er^3+^-doped YAG crystal with four-level ionic configuration. By taking advantage of the incoherent pumping process and the periodic spatial modulation of the driving field with standing wave pattern, a gain grating or hybrid (gain-phase) grating with high diffraction efficiency can be realized. We demonstrate that the incoherent pumping field and the probe detuning play important roles on the forming of the 1D and 2D gratings and their diffraction efficiency. By increasing the incoherent pumping rate and decreasing the probe detuning, we can significantly enhance the interference between the gain and phase gratings, and thereby improving the diffraction efficiency of the crystal grating. Furthermore, it is found that the diffraction efficiency of the crystal gratings is controllable by tuning the probe detuning and the intensity and detuning of the SW driving field or the concentration of Er^3+^ ion. More importantly, the probe energy of the diffraction side lobes around the zeroth diffraction order is comparable to that of the first-order diffraction for small driving intensity or large driving detuning. Moreover, our results also show that 1D and 2D crystal gratings exhibit different diffraction characteristics for the same optical parameters. Such crystal gratings, operating in the fiber communication band, may be more useful in optical communication and optical information processing.

## Model and Method

### Light-matter interactions in Er^3+^-doped YAG crystal

**Figure 1:**
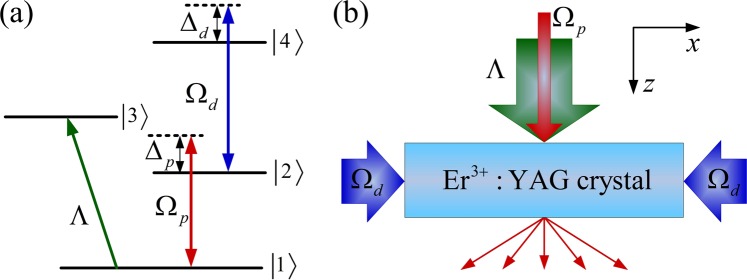
(**a**) Schematic of diagram of a four-level Er^3+^ ionic system interacting with probe, driving and incoherent pumping fields. (**b**) Sketch of the spatial configuration of the three laser beams with respect to the crystal and diffraction orders.

As schematically shown in Fig. 1(a), we consider a four-level Er^3+^ ionic system in an Er^3+^-doped YAG crystal. The designated states can be chosen as follows: $|1\rangle ={}^{4}{I}_{15/2}$, $|2\rangle ={}^{4}{I}_{13/2}$, $|3\rangle ={}^{4}{I}_{11/2}$ and $|4\rangle ={}^{4}{I}_{9/2}$. A weak probe field *E*_*p*_ with Rabi frequency Ω_*p*_ and an incoherent pumping field with an pumping rate *R* are applied to the transitions $\left|\,2\right\rangle \to \left|\,1\right\rangle$ and $\left|\,3\right\rangle \to \left|\,1\right\rangle$, respectively, while the transition $\left|\,4\right\rangle \to \left|\,2\right\rangle$ is driven by a strong driving field *E*_*d*_ with Rabi frequency Ω_*d*_. Here, we take the level $\left|\,1\right\rangle$ as the energy origin. In the Schrödinger picture and under the electric-dipole and rotating-wave approximations, the total Hamiltonian for the four-level Er^3+^ ionic system is given by

1 $$\begin{array}{rcl}H & = & \hslash {\omega }_{21}\left|\,2\right\rangle \left\langle 2\,\right|+\hslash ({\omega }_{32}+{\omega }_{21})\left|\,3\right\rangle \left\langle 3\,\right|+\hslash ({\omega }_{42}+{\omega }_{21})\left|\,4\right\rangle \left\langle 4\,\right|\\  &  & -\ \hslash \left({\Omega }_{p}{e}^{-i{\omega }_{p}t}\left|\,2\right\rangle \left\langle 1\right|+{\Omega }_{d}{e}^{-i{\omega }_{d}t}\left|\,4\right\rangle \left\langle 2\,\right|+H\,.\,c.\right),\end{array}$$

where *ω*_*ij*_ is the corresponding resonance frequency of the transition $\left|\,i\right\rangle \to \left|\,j\right\rangle$. We choose ${H}_{0}=\hslash {\omega }_{p}\left|\,2\right\rangle \left\langle 2\,\right|+\hslash ({\omega }_{p}+{\omega }_{c})\left|\,4\right\rangle \left\langle 4\,\right|$ as the free Hamiltonian. Using the transform formula ${H}_{I}={e}^{i{H}_{0}t/\hslash }(H-{H}_{0}){e}^{-i{H}_{0}t/\hslash }$, we obtain the interaction Hamiltonian in the interaction picture, which is written as

2 $${H}_{I}=-\,\hslash {\Delta }_{p}\left|\,2\right\rangle \left\langle 2\,\right|-\hslash ({\Delta }_{p}+{\Delta }_{c})\left|\,4\right\rangle \left\langle 4\,\right|-\hslash \left({\Omega }_{p}\left|\,2\right\rangle \left\langle 1\right|+{\Omega }_{d}\left|\,4\right\rangle \left\langle 2\,\right|+H\,.\,c.\right),$$

where Δ_*p*_ = *ω*_*p*_ − *ω*_21_ and Δ_*d*_ = *ω*_*d*_ − *ω*_42_ are the detunings of the probe and driving fields, respectively. Equation (2) describes the interaction between the coherent applied fields and Er3+ ions. It is worth noting that Eq. (2) can also be rewritten in a 4 × 4 matrix form:

3 $${H}_{I}=-\,\hslash (\begin{array}{cccc}0 & {\Omega }_{p}^{\ast } & 0 & 0\\ {\Omega }_{p} & {\Delta }_{p} & 0 & {\Omega }_{d}^{\ast }\\ 0 & 0 & 0 & 0\\ 0 & {\Omega }_{d} & 0 & {\Delta }_{p}+{\Delta }_{d}\end{array}).$$

The dynamics of the atomic system can be described by using the density matrix approach as

4 $$\frac{\partial \rho }{\partial t}=-\,\frac{i}{\hslash }\left[{H}_{I},\rho \right]+L\left[\rho \left(t\right)\right].$$

Here, the Liouvillian matrix *L*[*ρ*(*t*)] indicating the irreversible relaxation can be written as

5 $$L[\rho (t)]=\left(\begin{array}{cccc}{\sigma }_{11} & -{\gamma }_{21}{\rho }_{12} & -{\gamma }_{31}{\rho }_{13} & -{\gamma }_{41}{\rho }_{14}\\ -{\gamma }_{21}{\rho }_{21} & {\sigma }_{22} & -{\gamma }_{32}{\rho }_{23} & -{\gamma }_{42}{\rho }_{24}\\ -{\gamma }_{31}{\rho }_{31} & -{\gamma }_{32}{\rho }_{32} & -{\sigma }_{33} & -{\gamma }_{43}{\rho }_{34}\\ -{\gamma }_{41}{\rho }_{41} & -{\gamma }_{42}{\rho }_{42} & -{\gamma }_{43}{\rho }_{43} & -{\sigma }_{44}\end{array}\right),$$

where *σ*_11_ = *R*(*ρ*_33_ − *ρ*_11_) + Γ_21_*ρ*_22_ + Γ_31_*ρ*_33_ + Γ_41_*ρ*_44_, *σ*_22_ = Γ_32_*ρ*_33_ + Γ_42_*ρ*_44_ − Γ_21_*ρ*_22_, *σ*_33_ = *R*(*ρ*_33_ − *ρ*_11_) + (Γ_31_ + _32_)*ρ*_33_ − Γ_43_*ρ*_44_ and *σ*_44_ = (Γ_41_ + Γ_42_ + Γ_43_)*ρ*_44_. Γ_*ij*_ is the spontaneous-emission decay rate from the state $$\left|\,i\right\rangle $$ to the state $$\left|\,j\right\rangle $$, while *γ*_*ij*_ is the decay rate of the coherence between the states $$\left|\,i\right\rangle $$ and $$\left|\,j\right\rangle $$ (*i*, *j* = 1, 2, 3, 4; *i* > *j*), given by $${\gamma }_{21}=({\Gamma }_{21}+R+{\gamma }_{21}^{dph})/2$$, $${\gamma }_{31}=({\Gamma }_{31}+{\Gamma }_{32}+2R+{\gamma }_{31}^{dph})/2$$, $${\gamma }_{32}=({\Gamma }_{31}+{\Gamma }_{32}+{\Gamma }_{21}+R+{\gamma }_{32}^{dph})/2$$, $${\gamma }_{41}=({\Gamma }_{41}+{\Gamma }_{42}+{\Gamma }_{43}+R+{\gamma }_{41}^{dph})$$/2, $${\gamma }_{42}=({\Gamma }_{41}+{\Gamma }_{42}+{\Gamma }_{43}+{\Gamma }_{21}+{\gamma }_{42}^{dph})$$/2 and $${\gamma }_{43}=({\Gamma }_{41}+{\Gamma }_{42}+{\Gamma }_{43}$$$$+{\Gamma }_{31}+{\Gamma }_{31}+R+{\gamma }_{43}^{dph})$$/2. The Rabi frequencies of the probe and driving fields are defined by

6 $${\Omega }_{p}={\mu }_{21}{E}_{p}/2\hslash ,{\Omega }_{d}={\mu }_{42}{E}_{d}/2\hslash ,$$

where *μ*_*ij*_ is the electric-dipole matrix moment between level $\left|\,i\right\rangle$ and level $\left|\,j\right\rangle$, which can be calculated via the following equation^38,39^:

7 $$\begin{array}{rcl}{\mu }_{ij}^{2} & = & {\left|\left\langle \psi \right|er\left|{\psi }^{{\prime} }\right\rangle \right|}^{2}\\  & = & \frac{2}{2J+1}\frac{2}{2{J}^{{\prime} }+1}{e}^{2}\sum _{m=2,4,6}{\Omega }_{m}{| \left\langle 4{f}^{N}(\alpha SL)J\left|| {U}^{m}\right|| 4{f}^{N}({\alpha }^{{\prime} }{S}^{{\prime} }{L}^{{\prime} }){J}^{{\prime} }\right\rangle | }^{2},\end{array}$$

where Ω_*m*_(*m* = 2, 4, 6) represents the phenomenological intensity parameter, *J* and ${J}^{{\prime} }$ are the quantum numbers of angular momentum of the state $\left|\,i\right\rangle$ and $\left|\,j\right\rangle$, respectively. The factor 2 in Eq. (7) arose from the Kramers degenerate of the Stark levels of the Er^3+^ ion. The squared reduced matrix element ${| \left\langle 4{f}^{N}(\alpha SL)J\left|| {U}^{m}\right|| 4{f}^{N}({\alpha }^{{\prime} }{S}^{{\prime} }{L}^{{\prime} }){J}^{{\prime} }\right\rangle | }^{2}$ can be obtained^40^ and the spectral intensity parameters can be described by an empirical formula^41^

8 $$\begin{array}{ccc}{\Omega }_{m}(1{0}^{-20})=1.25{X}^{1/4}\exp [-0.4{(X-A)}^{2/3}]+B, &  & \end{array}$$

where *X* is the concentration of the doped Er^3+^ ions. *A* and *B* are empirical parameters: *A* = 1.0 and *B* = 0.33 for Ω_2_; *A* = 1.1 and *B* = 0.7 for Ω_4_; *A* = 1.4 and *B* = 0.59 for Ω_6_.

In the limit of weak probe field, a perturbation expansion method is used for deriving the analytical and steady-state solution for ${\rho }_{ij}^{l}(i,j=1,2,3,4)$. We take the expansions ${\rho }_{ij}={\sum }_{m=0}^{\infty }{\varepsilon }^{m}{\rho }_{ij}^{(m)}$ and ${\Omega }_{p}={\sum }_{n=1}^{\infty }{\varepsilon }^{n}{\Omega }_{p}^{(n)}$ and solve Eq. (4) order by order. At the zero order, we obtain non-zero density-matrix elements as

9 $$\begin{array}{rcl}{\rho }_{11}^{(0)} & = & \frac{{\kappa }_{1}{\Gamma }_{21}{\Gamma }_{31}+{\kappa }_{1}{\Gamma }_{21}{\Gamma }_{32}+{\kappa }_{3}{\Gamma }_{31}{\Gamma }_{41}+{\kappa }_{3}{\Gamma }_{32}{\Gamma }_{41}+{\kappa }_{3}{\Gamma }_{31}{\Gamma }_{43}}{{\kappa }_{1}{\kappa }_{2}+{\kappa }_{3}{\kappa }_{4}},\\ {\rho }_{22}^{(0)} & = & \frac{{\kappa }_{1}{\Gamma }_{32}R}{{\kappa }_{1}{\kappa }_{2}+{\kappa }_{3}{\kappa }_{4}},\\ {\rho }_{33}^{(0)} & = & \frac{{\kappa }_{1}{\Gamma }_{21}R+{\kappa }_{3}{\Gamma }_{41}R+{\kappa }_{3}{\Gamma }_{42}R}{{\kappa }_{1}{\kappa }_{2}+{\kappa }_{3}{\kappa }_{4}},\\ {\rho }_{44}^{(0)} & = & \frac{{\kappa }_{3}{\Gamma }_{32}R}{{\kappa }_{1}{\kappa }_{2}+{\kappa }_{3}{\kappa }_{4}},\end{array}$$

where $${\kappa }_{1}={\Gamma }_{41}+{\Gamma }_{42}+{\Gamma }_{43}+[2| {\Omega }_{d}{| }^{2}{\gamma }_{42}/({\gamma }_{42}^{2}+{\Delta }_{d}^{2})]$$, *κ*_2_ = Γ_21_Γ_31_ + Γ_21_Γ_32_ + 2Γ_21_*R* + Γ_32_*R*, $${\kappa }_{3}=2| {\Omega }_{d}{| }^{2}{\gamma }_{42}/({\gamma }_{42}^{2}+{\Delta }_{d}^{2})$$ and *κ*_4_ = Γ_31_Γ_41_ + Γ_32_Γ_41_ + Γ_31_Γ_43_ + Γ_32_*R* + 2Γ_41_*R* + 2Γ_43_*R*.

At the first order, we obtain

10 $${\rho }_{21}^{(1)}=\frac{i({\rho }_{11}^{(0)}-{\rho }_{22}^{(0)}){\Omega }_{p}-\frac{i| {\Omega }_{d}{| }^{2}({\rho }_{44}^{(0)}-{\rho }_{22}^{(0)}){\Omega }_{p}}{[{\gamma }_{41}-i({\Delta }_{p}+{\Delta }_{d})]({\gamma }_{42}-i{\Delta }_{d})}}{{\gamma }_{21}-i{\Delta }_{p}+\frac{| {\Omega }_{d}{| }^{2}}{{\gamma }_{41}-i({\Delta }_{p}+{\Delta }_{d})}}.$$

Therefore, the probe susceptibility *χ*_*p*_, can be written as

11 $${\chi }_{p}=\frac{{N}_{0}| {\mu }_{21}{| }^{2}}{{\epsilon }_{0}\hslash {\Omega }_{p}}{\rho }_{21}^{(1)}=\frac{{N}_{0}| {\mu }_{21}{| }^{2}}{{\epsilon }_{0}\hslash \Gamma }\chi =\frac{{N}_{0}| {\mu }_{21}{| }^{2}}{{\epsilon }_{0}\hslash \Gamma }\cdot \frac{i({\rho }_{11}^{(0)}-{\rho }_{22}^{(0)})-\frac{i| {\Omega }_{d}{| }^{2}({\rho }_{44}^{(0)}-{\rho }_{22}^{(0)})}{[{\gamma }_{41}-i({\Delta }_{p}+{\Delta }_{d})]({\gamma }_{42}-i{\Delta }_{d})}}{{\gamma }_{21}-i{\Delta }_{p}+\frac{| {\Omega }_{d}{| }^{2}}{{\gamma }_{41}-i({\Delta }_{p}+{\Delta }_{d})}}\Gamma ,$$

where *N*_0_ denotes the number of doped ions per unit volume. Note that the real and imaginary parts of probe susceptibility *χ*_*p*_ represent the dispersion and absorption-gain, respectively.

### Fraunhofer diffraction of 1D EIG

It can be seen from Eq. (11) that both the real and imaginary parts of the probe susceptibility *χ*_*p*_ depend on the intensity of the driving field. The space-dependent driving field can result in the spatial modulation of the dispersion and absorption-gain for probe field. In this case, the Er^3+^-doped YAG crystal can be treated as an EIG. For 1D EIG, the 1D space-dependent driving field Ω_*d*_(*x*) is a SW field, which can be written as

12 $${\Omega }_{d}(x)={\Omega }_{d0}\sin (\pi x/\Lambda ),$$

where 2Λ is the spatial period of the SW field. In this situation, 1D EIG can diffract the probe beam propagating in the *z* direction into different diffraction directions. Under the slowly varying envelope approximation and in the steady-state regime, the propagation of the probe field is described by the reduced wave equation as

13 $$\frac{\partial {E}_{p}}{\partial z}=i\frac{\pi }{{\epsilon }_{0}{\lambda }_{p}}P=i\frac{\pi }{{\lambda }_{p}}{\chi }_{p}{E}_{p},$$

where *λ*_*p*_ is the wavelength of the probe beam. Equation (13) can be rewritten as $\partial {E}_{p}/\partial {z}^{{\prime} }=i\chi {E}_{p}$, where ${z}^{{\prime} }=(\pi {N}_{0}| {\mu }_{21}{| }^{2})\cdot z/({\epsilon }_{0}\hslash {\lambda }_{p}\Gamma )$. Note that ${z}^{{\prime} }$ is a dimensionless variable by setting *ζ* = (*ϵ*_0_*ℏλ*_*p*_Γ)/(*πN*_0_|*μ*_21_|^2^) as the unit of *z*.

We assume that the interaction length between Er^3+^ ions and probe field along the *z* direction, i.e., the thickness of the thin Er^3+^-doped YAG crystal, is *L*. Thus, the transmission function, which is defined as the ratio of the output field amplitude to the input field amplitude, can be given by

14 $$T(x)={e}^{-Im[\chi (x)]L}{e}^{iRe[\chi (x)]L},$$

where |*T*(*x*)| = *e*^−*Im*[*χ*(*x*)]*L*^ and Φ(*x*) = *Re*[*χ*(*x*)]*L* are the amplitude and phase of 1D transmission function, respectively. Such a grating is the superposition of an amplitude grating and a phase grating.

By 1D Fourier transform of *T*(*x*), we can obtain 1D Fraunhofer diffraction-intensity function:

15 $${I}_{p}(\theta )=| F(\theta ){| }^{2}\frac{{\sin }^{2}(M\pi \Lambda \sin \theta /{\lambda }_{p})}{{M}^{2}{\sin }^{2}(\pi \Lambda \sin \theta /{\lambda }_{p})},$$

where *θ* indicates the diffraction angle with respect to the *z* direction and *M* represents the number of spatial periods of the atomic grating illuminated by the probe beam. *F*(*θ*) is the Fraunhofer diffraction of a single space period Λ, which is given by

16 $$F(\theta )=\frac{1}{\Lambda }{\int }_{0}^{\Lambda }T(x){e}^{-i2\pi x\sin \theta /{\lambda }_{p}}dx.$$

In particular, if the condition of $\sin {\theta }_{m}=m{\lambda }_{p}/\Lambda$ is satisfied, the diffraction intensity *I*_*m*_ along the *m*-order diffraction direction can be calculated by ${I}_{m}=| F({\theta }_{m}){| }^{2}=(1/\Lambda ){\int }_{0}^{\Lambda }T(x){e}^{-i2\pi mx/\Lambda }dx$.

### Fraunhofer diffraction of 2D EIG

For 2D EIG, the 2D space-dependent driving field ${\Omega }_{d}({x}^{{\prime} },{y}^{{\prime} })$ is a superposition of two orthogonal SW fields with the same frequency along the ${x}^{{\prime} }$ and ${y}^{{\prime} }$ directions, i.e., ${\Omega }_{d}({x}^{{\prime} },{y}^{{\prime} })={\Omega }_{d0}[\sin (\pi {x}^{{\prime} }{/\Lambda }^{{\prime} })+\sin (\pi {y}^{{\prime} }{/\Lambda }^{{\prime} })]$, where ${2\Lambda }^{{\prime} }$ is the period of the two SW fields. It should be noted that ${x}^{{\prime} }{y}^{{\prime} }$ coordinates are obtained by rotating the *xy* coordinates counterclockwise 45 degrees, and then ${x}^{{\prime} }=\sqrt{2}x/2+\sqrt{2}y/2$ and ${y}^{{\prime} }=-\,\sqrt{2}x/2+\sqrt{2}y/2$. Thus, in the *xy* coordinates, the 2D driving field can be rewritten as

17 $${\Omega }_{d}(x,y)={\Omega }_{d0}[\sin (\pi (x+y)/\Lambda )+\sin (\pi (-x+y)/\Lambda )],$$

in which ${2\Lambda }^{{\prime} }=\sqrt{2}\Lambda$ is selected. In this case, the dispersion and absorption-gain can be periodically modulated along the *x* and *y* directions with the period Λ. Therefore,the 2D transmission function *T*(*x*, *y*) can be written as

18 $$T(x,y)={e}^{-Im[\chi (x,y)]L}{e}^{iRe[\chi (x,y)]L},$$

where |*T*(*x*, *y*)| = *e*^−*Im*[*χ*(*x*, *y*)]*L*^ and Φ(*x*, *y*) = *Re*[*χ*(*x*, *y*)]*L* are the amplitude and phase of the transmission function, respectively.

By 2D Fourier transform of *T*(*x*, *y*), we can obtain 2D Fraunhofer diffraction-intensity function:

19 $${I}_{p}({\theta }_{x},{\theta }_{y})=| F({\theta }_{x},{\theta }_{y}){| }^{2}\frac{{\sin }^{2}({M}_{x}\pi \Lambda \sin {\theta }_{x}/{\lambda }_{p})}{{M}_{x}^{2}{\sin }^{2}(\pi \Lambda \sin {\theta }_{x}/{\lambda }_{p})}\frac{{\sin }^{2}({M}_{y}\pi \Lambda \sin {\theta }_{y}/{\lambda }_{p})}{{M}_{y}^{2}{\sin }^{2}(\pi \Lambda \sin {\theta }_{y}/{\lambda }_{p})},$$

where *θ*_*x*(*y*)_ indicates the diffraction angle with respect to the *z* direction in the *x*(*y*) − *z* plane and *M*_*x*(*y*)_ represents the number of spatial periods of the grating along the *x*(*y*) direction. The Fraunhofer diffraction *F*(*θ*_*x*_, *θ*_*y*_) of a single space period Λ in 2D space is given by

20 $$F({\theta }_{x},{\theta }_{y})=\frac{1}{{\Lambda }^{2}}{\int }_{0}^{\Lambda }dx{e}^{-i2\pi x\sin {\theta }_{x}/{\lambda }_{p}}{\int }_{0}^{\Lambda }T(x,y){e}^{-i2\pi y\sin {\theta }_{y}/{\lambda }_{p}}dy.$$

Here, when both $$\sin {\theta }_{x}^{m}=m{\lambda }_{p}/\Lambda $$ and $$\sin {\theta }_{y}^{n}=n{\lambda }_{p}/\Lambda $$ are satisfied, we can obtain the diffraction intensity *I*_(*m*, *n*)_ along the (*m*, *n*)th-order diffraction direction as $${I}_{(m,n)}=| F({\theta }_{x}^{m},{\theta }_{y}^{n}){| }^{2}=(1/{\Lambda }^{2}){\int }_{0}^{\Lambda }{e}^{-i2\pi mx/\Lambda }dx{\int }_{0}^{\Lambda }T(x,y)$$$${e}^{-i2\pi ny/\Lambda }dy$$.

## Experimental realization

For the experimental realization, we would like to mention some points of the Er^3+^-doped YAG crystal for the present study, which are given as follows:

(I) Based on the experimental results^41,42^, we can get the spontaneous-emission decay rate Γ_*ij*_ of the Er^3+^ ions for different concentrations of Er^3+^ ion at room temperature. For simplicity, all the parameters have been scaled by Γ = 239.1*s*^−1^. So it is reasonable that we choose the parameters as Γ_21_ = Γ, Γ_31_ = 0.8Γ, Γ_32_ = 10Γ, Γ_41_ = 0.86Γ, Γ_42_ = 0.29Γ, Γ_43_ = 0.04Γ for 0.52 at . % Er^3+^ ion and Γ_21_ = 1.08Γ, Γ_31_ = 0.91Γ, Γ_32_ = 9.89Γ, Γ_41_ = 0.88Γ, Γ_42_ = 0.32Γ, Γ_43_ = 0.07Γ for 0.79 at . % Er^3+^.
(II) According to the experimental result^43^, we have found that the dephasing time of Er^3+^-doped YAG crystal with an Er^3+^ concentration of 0.1%, *T*_2_ = 75 *μs* on the transition ^4^*I*_15∕2_ → ^4^*I*_13∕2_ of Er^3+^ at 1526.97 nm, the homogeneous linewidth Γ_*h*_ = 4286 Hz. Thus, it is reasonable for us to estimate the dephasing decay rate as ${\gamma }_{21}^{dph}={\gamma }_{31}^{dph}={\gamma }_{32}^{dph}={\gamma }_{41}^{dph}={\gamma }_{42}^{dph}={\gamma }_{43}^{dph}=15\Gamma$.
(III) Based on Eqs. (7) and ((8)), we obtain *μ*_42_ = 2.662 × 10^−32^ Cm for 0.52 at . % Er^3+^ ion and *μ*_42_ = 2.799 × 10^−32^ Cm for 0.79 at . % Er^3+^ ion.

## Results and Discussions

In this section, we focus on analyzing the Fraunhofer diffraction characteristics of the probe beam by adjusting the controllable optical parameters of 1D and 2D EIGs realized in an Er^3+^-doped YAG crystal. Before presenting the numerical results, we first give the creditable evaluation of the numerical computation. Our numerical calculation is based on MATLAB R2015b software. We use the embedded FFT package to make 1D and 2D fast Fourier transform of the transmission function *T*(*x*) and select Λ∕40 as the step size of Fourier transform. Continuing to increase the sample points and decrease the step size would not result in the change of the diffraction spectra, which can prove the validity of our numerical computation.

**Figure 2:**
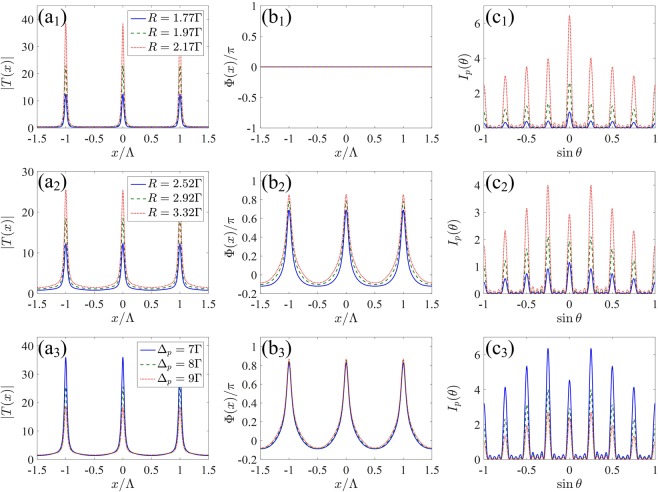
(**a**_1_–**a**_3_) The amplitude |*T*(*x*)| and (**b**_1_–**b**_3_) the phase Φ(*x*)/*π* of the transmission function as a function of *x*, and (**c**_1_–**c**_3_) Fraunhofer diffraction intensity *I*_*p*_(*θ*) as a function of $\sin \theta$ for various *R* and Δ_*p*_. (**a**_1_,**b**_1_,**c**_1_) Δ_*p*_ = 0; (**a**_2_,**b**_2_,**c**_2_) Δ_*p*_ = 8Γ; (**a**_3_,**b**_3_,**c**_3_) *R* = 3.32Γ. Other parameters are Ω_*d*0_ = 10Γ, Δ_*d*_ = 0, *M* = 5, Λ/*λ*_*p*_ = 4 and *L* = 140*ζ*.

**Figure 3:**
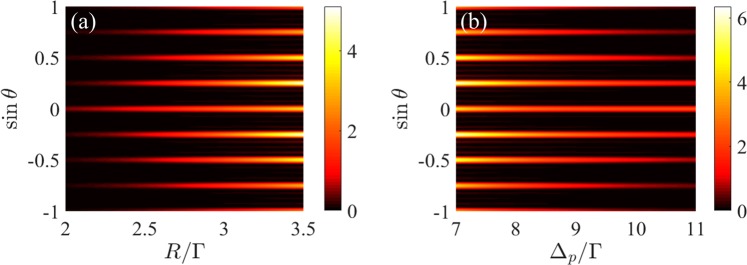
Fraunhofer diffraction spectra of 1D grating as a function of (**a**) the incoherent pumping rate *R* with Δ_*p*_ = 8Γ and (**b**) the probe detuning Δ_*p*_ with *R* = 3.32Γ. Other parameters are the same as in Fig. 2.

For the case of 1D EIG, we first examine in Fig. 2 the influence of incoherent pumping rate *R* and probe detuning Δ_*p*_ on the Fraunhofer diffraction of the crystal grating. Here, we select Er^3+^:  YAG crystal containing 0.52 at . % concentrations of Er^3+^ ion. Typical curves of the amplitude |*T*(*x*)| of the transmission function are shown in Fig. 2(a_1_–a_3_) for various *R* and Δ_*p*_. It is obvious that the maxima of the amplitude |*T*(*x*)| are always located at the nodes of the SW driving field Ω_*d*_(*x*). The corresponding curves of the phase Φ(*x*) of the transmission function are also plotted in Fig. 2(b_1_–b_3_). When Δ_*p*_ = 0, the amplitude |*T*(*x*)| is greatly improved with the increase of *R* from 1.77Γ to 2.17Γ because of the enhancement of probe gain in the incoherent pump process [see Fig. 2(a_1_)], while the phase Φ(*x*), which is unaffected by the change of *R*, always equals to zero due to the zero dispersion in the resonant light-matter interaction [see Fig. 2(b_1_)]. That is to say, only amplitude modulation takes place and the crystal grating is a pure gain grating. As shown in Fig. 2(c_1_), the diffraction intensities in all diffraction orders are remarkably improved via increasing the incoherent pumping rate, but the central principle maximum (zeroth-order diffraction) always dominates due to the limitation of amplitude grating^8^. As Δ_*p*_ is increased from 0 to 8Γ, the space-dependent dispersion exists and the phase *Φ*(*x*) presents an inhomogeneous distribution over one space period. In this case, the crystal grating becomes a hybrid grating. As shown in Fig. 2(a_2_,b_2_), both the amplitude |*T*(*x*)| and the phase modulation depth Δ*Φ*, i.e., $\Delta \Phi =\max [\Phi (x)]-\min [\Phi (x)]$, increase with the increase of *R* from 2.52Γ to 3.32Γ. As we know, the increase of the amplitude modulation can enhance the intensities of the diffraction fields, while the increase of the phase modulation can improve the ratio of the diffraction intensities in the high diffraction directions. In this case, the hybrid grating can be treated as a superposition of a gain grating and a phase grating^19^, increasing the amplitude and phase modulations can enhance the interference between the gain and phase gratings, and thereby leading to the improvement of the diffraction efficiencies and more probe energy being diffracted into high diffraction orders. It is worth noting that the hybrid grating requires stronger incoherent pumping rate than the pure gain grating under the condition of achieving the same first-order diffraction intensity [see red dotted lines in Fig. 2(c_1_,c_2_)]. For a fixed incoherent pumping rate, i.e, *R* = 3.32Γ, when the probe detuning Δ_*p*_ is varied from 7Γ to 9Γ, the amplitude |*T*(*x*)| is decreased but the phase Φ(*x*) with Δ*Φ* ≃ *π* remains almost unchanged [see Fig. 2(a_3_,b_3_)]. The decrease of the amplitude modulation weakens the interference of the gain and phase gratings. Thus, the diffraction intensities of the diffraction fields decrease. However, the first-order diffraction peak is always highest owing to unchanged phase modulation [see Fig. 2(c_3_)]. In order to gain overall view of the effect of the incoherent pumping rate *R* and probe detuning Δ_*p*_, we present the corresponding evolutions of the diffraction spectra *I*_*p*_(*θ*) with the increase of *R* and Δ_*p*_ in Fig. 3, respectively. It is found that the diffraction efficiency of the crystal grating increases monotonically as *R* increases in the range of [2Γ, 3.5Γ] or *Δ*_*p*_ decreases in the range of [7Γ, 11Γ] [see Fig. 3(a,b)].

To obtain the corresponding power of the incoherent pumping field, the incoherent pumping rate *R* can be written as *R* = *σ*_13_*I*_*ip*_∕*hν*_*ip*_, where *ν*_*ip*_ and *I*_*ip*_ are the frequency and light intensity of the incoherent pumping field, respectively. *h* is Planck constant and *σ*_13_ is the pump absorption section of Er^3+^ ion. The absorption cross-section at 967 nm pump wavelength (*ν*_*ip*_ = 3.1 × 10^14^*s*^−1^) is 2.8 × 10^−20^ cm^2^ ^44,45^. Thus, the light intensity *I*_*ip*_ of the incoherent pumping field can be calculated for a certain value of *R*. Then, we can obtain the power of the incoherent pumping field via the formula *P* = *AI*_*ip*_, where *A* is the cross-sectional area of the incoherent pumping field. If the laser beam is focused into a spot with a diameter 0.1 mm, this requires the laser power of the incoherent pumping field arrives at *P* = 348 mW for *R* = 2.52Γ and *P* = 403 mW for *R* = 2.92Γ. It is obvious that the incoherent pumping field has surpassed the threshold power of the Er^3+^-doped YAG crystal. In addition, we assume that the incident probe field is sufficiently weak and the interaction length is relatively short. Therefore, our scheme satisfies small-signal model without considering the saturation effect. As for the SW driving field, the selected Rabi frequency, i.e., Ω_*d*0_ = 10Γ, is slightly larger than the selected incoherent pumping rate *R*. One can readily evaluate that the power of the SW driving field is higher the power of the incoherent pumping field.

**Figure 4:**
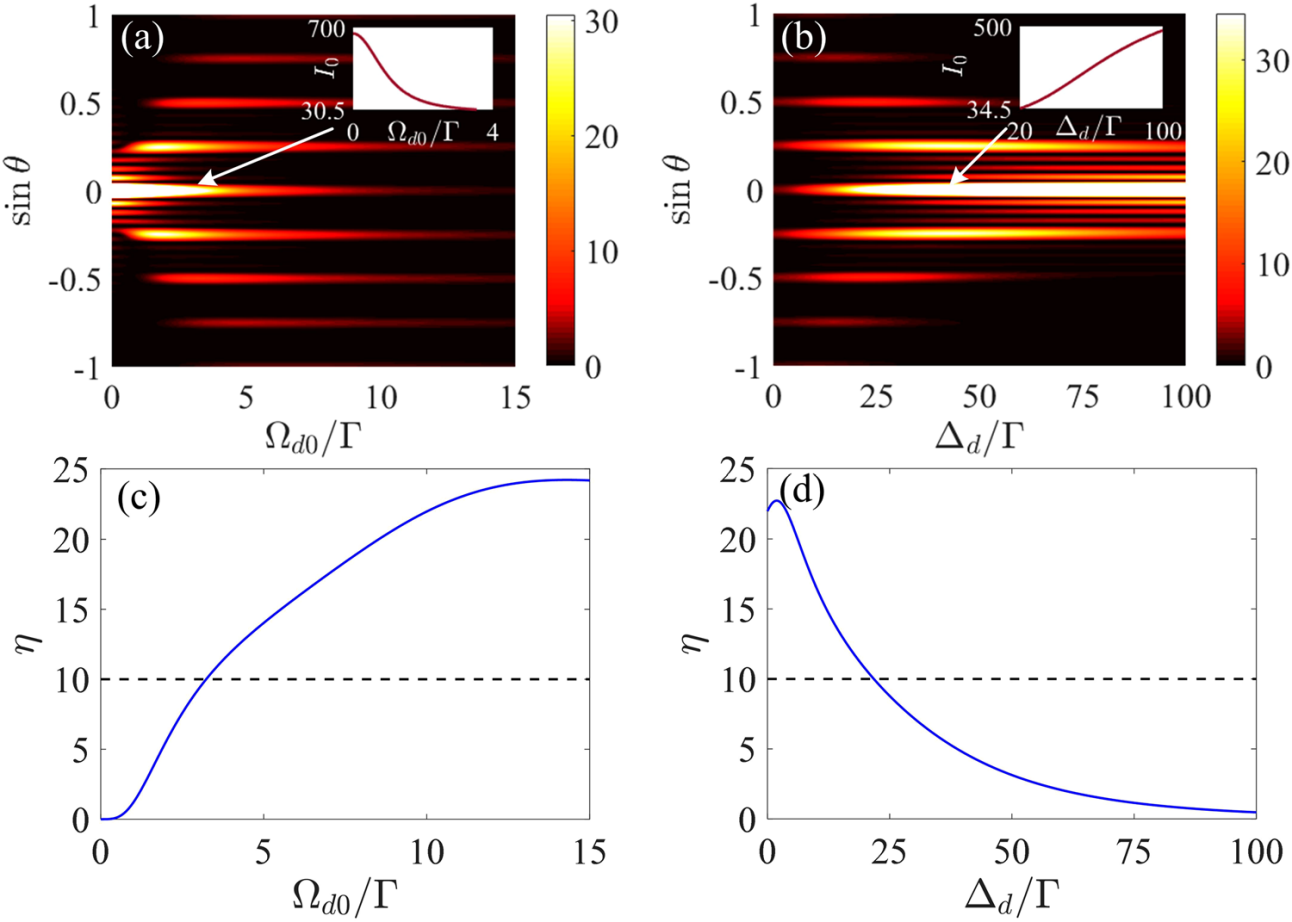
(**a**,**b**) Fraunhofer diffraction spectra and (**c**,**d**) diffraction contrast *η* of 1D grating as a function of (**a**,**c**) the intensity Ω_*d*0_ with Δ_*d*_ = 0 and (**b**,**d**) the detuning Δ_*d*_ with Ω_*d*0_ = 10Γ of the SW driving field. The insets in (**a**,**b**) show the diffraction intensity of the truncated zeroth-order diffraction field versus Ω_*d*0_ and Δ_*d*_, respectively. Other parameters are the same as in Fig. 2 except for *R* = 3.32Γ and Δ_*p*_ = 8Γ.

We then examine in Fig. 4 how the diffraction distribution of the crystal grating depends on the intensity and detuning of the SW driving field. In Fig. 4(a,b), the extremely large zeroth-order diffraction peaks are truncated and the corresponding intensity of the truncated zeroth-order diffraction field is shown as insets to clearly demonstrate the progress for various Ω_*d*0_ and Δ_*d*_. Figure 4(a) shows the effect of the intensity Ω_*d*0_ on Fraunhofer diffraction patterns. With the increase of Ω_*d*0_, the zeroth-order diffraction field decreases monotonically, while the diffraction fields in the high-order directions increase firstly and then decrease. In other words, there are optimal values of Ω_*d*0_ for which the high-order diffraction intensities reach their maxima. In Fig. 4(b), the effect of the detuning Δ_*d*_ on the diffraction patterns is presented. It can be seen that the high-order diffraction fields also increase firstly and then decrease as Δ_*d*_ increases, which is similar to the result in Fig. 4(a), while the diffraction intensity in the central principle maximum increases monotonically. Therefore, one can control the diffraction of the crystal grating by varying the intensity and detuning of the SW driving field. Direct comparison of Fig. 4(a,b) implies that the diffraction side lobes around the zeroth-order diffraction peak have the same evolution trend with the zeroth-order diffraction intensity. More importantly, the probe energy of the diffraction side lobes is comparable to that of the first-order diffraction for small driving intensity or large driving detuning, which would hamper the application of the first-order diffraction component. To choose the suitable parameters to realize the high diffraction efficiency of the first-order diffraction with suppressed diffraction side lobes, we define a “diffraction contrast *η*”, which is the intensity ratio of the first-order diffraction to the diffraction side lobe around the central principle maximum, i.e., *η* = *I*_1_∕*I*_*sl*_. The high performance grating can be obtained when the diffraction contrast *η* exceeds 10 (i.e., *η* ≥ 10). It is obvious that the diffraction contrast *η* ≥ 10 when Ω_*d*0_ ≥ 3.24Γ [see Fig. 4(c)] or Δ_*d*_ ≤ 21.7Γ [see Fig. 4(d)]. From Fig. 4(a,b), we can find that, in the high performance region, the intensity of the first-order diffraction field reaches its maximal value, i.e., *I*_1_ = 21.32 at Ω_*d*0_ = 3.24Γ and *I*_1_ = 22.85 at Δ_*d*_ = 21.7Γ. In this situation, *Ω*_*d*0_ = 3.24Γ and *Δ*_*d*_ = 21.7Γ are the corresponding optimal parameters to realize the optimal performance of the crystal grating.

**Figure 5:**
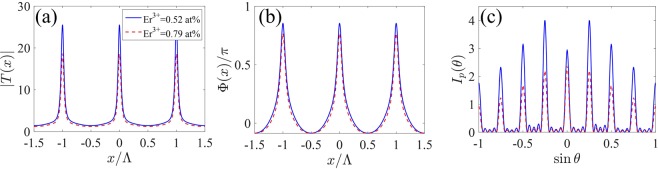
(**a**) The amplitude |*T*(*x*)| and (**b**) the phase Φ(*x*)∕*π* of the transmission function as a function of *x*, and (**c**) Fraunhofer diffraction intensity *I*_*p*_(*θ*) as a function of $\sin \theta$ for different concentrations of Er^3+^ ion. Other parameters are *R* = 3.32Γ, Δ_*d*_ = 0 and Δ_*p*_ = 8Γ.

It has been found that the concentration of Er^3+^ ion can greatly influence the optical properties of Er^3+^-doped YAG crystal^32,37^. In the following, we investigate the effect of the concentration of Er^3+^ ion on the diffraction characteristics of the crystal grating in Fig. 5. The concentration of Er^3+^ ion in Er^3+^-doped YAG crystal greatly affects the electric dipole moment *μ*_*ij*_. We keep the intensity *E*_*d*0_ of the standing-wave driving field constant. When *Ω*_*d*0_ = 10Γ for 0.52 at . % Er^3+^ ion concentration, we can obtain Ω_*d*0_ = 10.51Γ for 0.79 at. % Er^3+^ ion concentration. As shown in Fig. 5(a,b), both the amplitude |*T*(*x*)| and the phase modulation depth ΔΦ decrease with the increase of the Er^3+^ ion concentration from 0.52% to 0.79%. The decrease of both the amplitude and phase modulation reduces the interference between the gain and phase gratings. As a result, the diffraction efficiency of the grating is decreased and the diffraction energy is concentrated into the central principle maximum[see Fig. 5(c)]. These results offer us another controllable parameter to manipulate the diffraction behaviors of the crystal grating.

**Figure 6:**
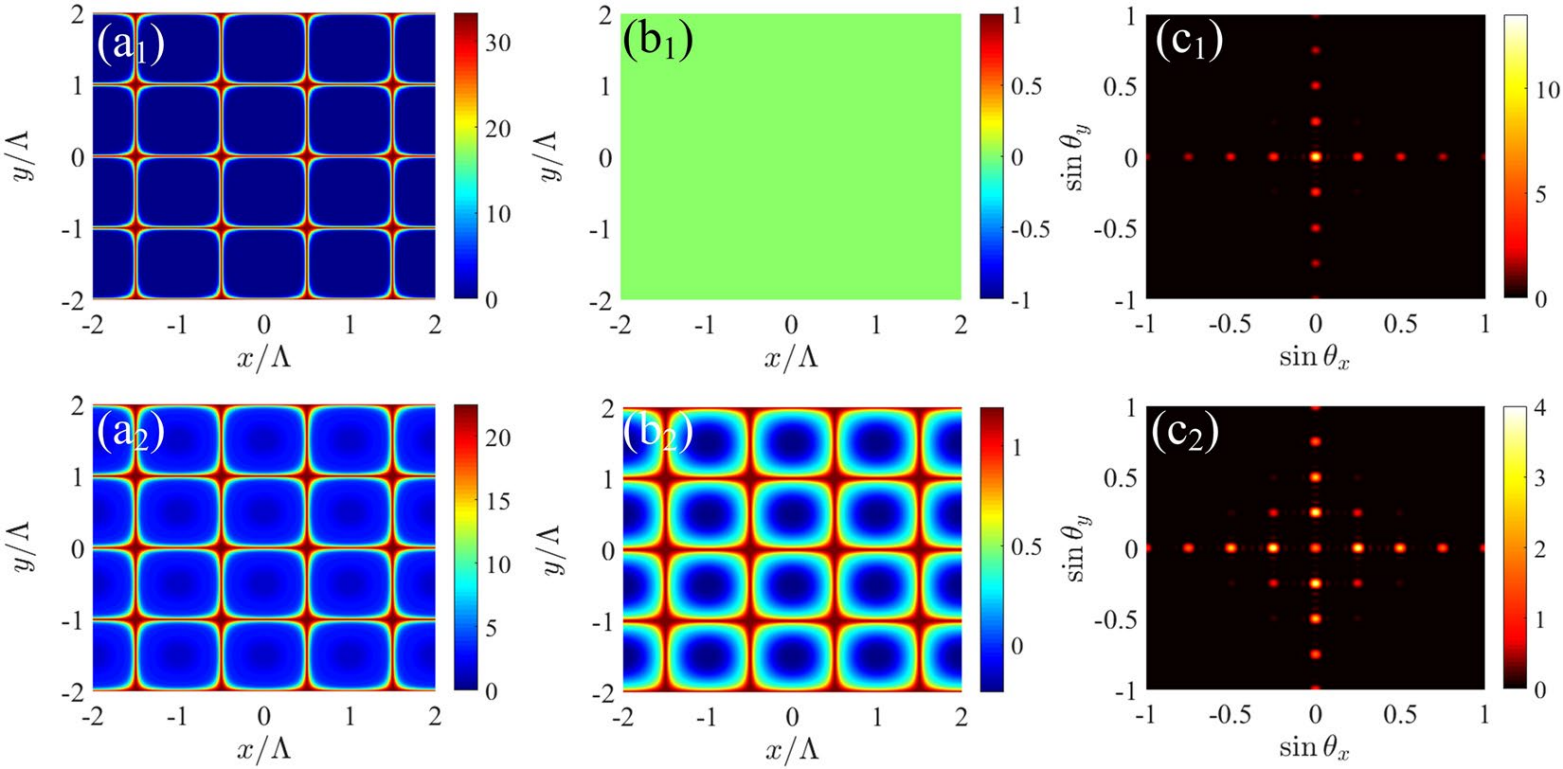
(**a**_1_,**a**_2_)The amplitude |*T*(*x*, *y*)| and (**b**_1_,**b**_2_) the phase Φ(*x*, *y*)/*π* of 2D transmission function as a function of (*x*, *y*), and (**c**_1_,**c**_2_) Fraunhofer diffraction intensity *I*_*p*_(*θ*_*x*_, *θ*_*y*_) as a function of $(\sin {\theta }_{x},\sin {\theta }_{y})$ for different *R* and Δ_*p*_. (**a**_1_,**b**_1_,**c**_1_) *R* = 1.833Γ and Δ_*p*_ = 0; (**a**_2_,**b**_2_,**c**_2_) *R* = 3.91Γ and Δ_*p*_ = 12Γ. Other parameters are Ω_*d*0_ = 8Γ, Δ_*d*_ = 0, *M*_*x*_ = *M*_*y*_ = 5, Λ/λ_*p*_ = 4 and *L* = 180*ζ*.

**Figure 7:**
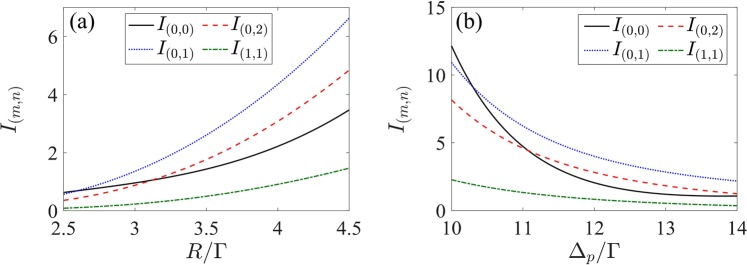
Fraunhofer diffraction intensities of 2D grating for some diffraction orders as a function of (**a**) the incoherent pumping rate *R* with Δ_*p*_ = 12Γ and (**b**) the probe detuning Δ_*p*_ with *R* = 3.91Γ. Other parameters are the same as in Fig. 6.

Let us now investigate the diffraction characteristics of 2D EIG. We also select Er^3+^:  YAG crystal containing 0.52 at . % concentrations of Er^3+^ ion. Such a grating can be realized when the space-dependent driving field is a superposition of two orthogonal SW fields [see Eq. (17)]. Similar to the diffraction of 1D grating, the incoherent pumping rate *R* and probe detuning Δ_*p*_ also play important roles in the energy distribution of different diffraction orders in 2D crystal grating. Figure 6 shows the influence of *R* and Δ_*p*_ on the transmission function and Fraunhofer diffraction patterns of the 2D grating. In the case of *R* = 1.833Γ and Δ_*p*_ = 0, the maxima of the amplitude |*T*(*x*, *y*)| are localized at the position (*x*, *y*), where *x* = (0.5 ± *m*) ⋅ Λ and *y* = ± *n* ⋅ Λ (*m*, *n* are integers), but the phase Φ(*x*, *y*) is zero [see Fig. 6(a_1_,b_1_)]. In this situation, the grating is a 2D pure gain grating. It can be seen that the most portion of probe energy is diffracted into the (± *m*, 0)- and (0, ± *n*)-order diffraction directions and the (0, 0)-order diffraction field dominates [see Fig. 6(c_1_)]. Here, the diffraction intensity in the first diffraction order, i.e., (± 1, 0) and (0, ± 1) orders, can arrive at 4. When *R* = 3.91Γ and Δ_*p*_ = 12Γ, as shown in Fig. 6(a_2_,b_2_), the maxima of the phase Φ(*x*, *y*) are localized at the positions, where the amplitude |*T*(*x*, *y*)| is maximal. Although the amplitude |*T*(*x*, *y*)| of the transmission function reduces in comparison with the case shown in Fig. 6(a_1_), but the approximate *π* phase modulation depth results in more probe energy being diffracted into the high-order diffraction directions. In this case, the diffraction intensities of the (± 1, 0)- and (0, ± 1)-order diffraction fields can also reach 4 [see Fig. 6(c_2_)]. To see more details, the evolutions of the diffraction intensities in the (0, 0), (0, 1), (0, 2) and (1, 1) diffraction orders with the incoherent pumping rate *R* and probe detuning Δ_*p*_ are plotted in Fig. 7. Similar to the 1D case, the studied four diffraction fields increase monotonically as *R* increases or Δ_*p*_ decreases and the (0, 1)- and (0, 2)-order diffraction intensities exceed the (0, 0)-order diffraction intensity for large *R* or Δ_*p*_ [see Fig. 7(a,b)]. Therefore, it can be concluded that the location of the maximal diffraction field of 2D grating can be manipulated via adjusting the values of *R* and Δ_*p*_.

**Figure 8:**
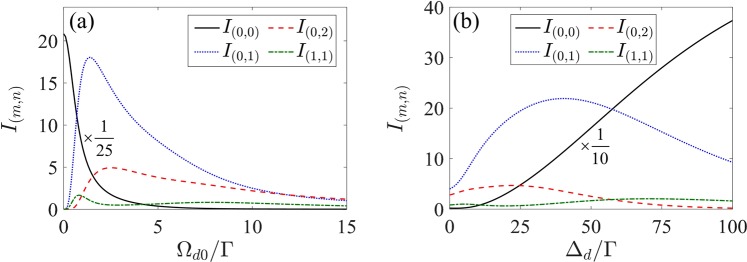
Fraunhofer diffraction intensities of 2D grating for some diffraction orders as a function of (**a**) the intensity Ω_*d*0_ with Δ_*d*_ = 0 and (**b**) the detuning Δ_*d*_ with Ω_*d*0_ = 8Γ of the SW driving field. Other parameters are the same as in Fig. 6 except for *R* = 3.91Γ and Δ_*p*_ = 12Γ.

We further examine in Fig. 8 the influence of the 2D space-dependent driving field on the diffraction of 2D grating. The evolutions of the diffraction intensities in the (0, 0), (0, 1), (0, 2) and (1, 1) diffraction orders with the intensity Ω_*d*0_ and the detuning Δ_*d*_ of the driving field are plotted in Fig. 8(a,b), respectively. It is found that increasing Ω_*d*0_ or decreasing Δ_*d*_ can lead to the reduction of the (0, 0)-order diffraction intensity but the diffraction intensities in the (0, 1) and (0, 2) diffraction orders increase firstly and then decrease. These trends are similar to the 1D cases shown in Fig. 4(a,b). However, unlike the (0, 1)- and (0, 2)-order diffraction fields, the (1, 1)-order diffraction field shows fluctuation in the diffraction intensity with respect to Ω_*d*0_ and Δ_*d*_.

**Figure 9:**
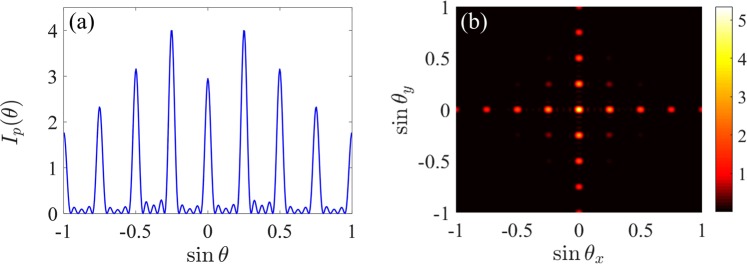
Fraunhofer diffraction spectra of (**a**) 1D and (**b**) 2D gratings for *R* = 3.32Γ and *M* = *M*_*x*_ = *M*_*y*_ = 5. Other parameters are the same as in Fig. 2(c_2_).

Finally, we examine in Fig. 9 what will happen when the same system parameters are selected for both 1D and 2D gratings? Here, we select the same parameters in Fig. 2(c_2_) except *R* = 3.32Γ and *M* = *M*_*x*_ = *M*_*y*_ = 5. One can find from Fig. 9(a,b) that the first-order diffraction field dominates in the diffraction of 1D grating, while the (0, 0)-order diffraction field is maximal in the diffraction of 2D grating. Meanwhile, the first-order diffraction intensity, i.e., *I*_1_ = 4, is larger than the (0, 1)-order diffraction intensity, i.e., *I*_(0, 1)_ = 3.32. These results indicate that 1D and 2D EIGs exhibit different diffraction characteristics for the same optical parameters.

In summary, we have theoretically investigated the Fraunhofer diffraction of 1D and 2D EIGs realized in Er^3+^-doped YAG crystal. In the presence of the incoherent pumping process, the induced spatial gain modulation without or with phase modulation results in the generation of the gain or hybrid grating, where the high diffraction intensities are achievable in the high-order diffraction directions. It is demonstrated that increasing the incoherent pumping rate and decreasing the probe detuning can significantly improve the diffraction efficiencies of the 1D and 2D crystal gratings. We give a suitable physical interpretation for the diffraction behaviors via the interference of the gain and phase gratings. Furthermore, it is found that the diffraction intensity of each diffraction field is also controllable by tuning the intensity and detuning of the SW driving field or the concentration of Er^3+^ ion. More importantly, the probe energy of the diffraction side lobes around the central principal maximum is comparable to that of the first-order diffraction field for small driving intensity or large driving detuning, which would limit the use of the first-order diffraction component. Based on this situation, we find the suitable optical parameters to realize the optimal performance of the grating. Finally, we note that the transition ${}^{4}I_{15/2}\to {}^{4}I_{13/2}$ driven by weak probe field coincides with the third transparency-window of the optical fiber. Therefore, our scheme may provide the possibility for the active all-optical control of optical switching, routing and storage in communication wavelengths.
